# A Prominent Role of Interleukin-18 in Acetaminophen-Induced Liver Injury Advocates Its Blockage for Therapy of Hepatic Necroinflammation

**DOI:** 10.3389/fimmu.2018.00161

**Published:** 2018-02-08

**Authors:** Malte Bachmann, Josef Pfeilschifter, Heiko Mühl

**Affiliations:** ^1^Pharmazentrum Frankfurt/ZAFES, University Hospital Goethe-University Frankfurt am Main, Frankfurt am Main, Germany

**Keywords:** interleukin-18, IL-18BP, interleukin-1, acetaminophen, liver injury, inflammation

## Abstract

Acetaminophen [paracetamol, *N*-acetyl-*p*-aminophenol (APAP)]-induced acute liver injury (ALI) not only remains a persistent clinical challenge but likewise stands out as well-characterized paradigmatic model of drug-induced liver damage. APAP intoxication associates with robust hepatic necroinflammation the role of which remains elusive with pathogenic but also pro-regenerative/-resolving functions being ascribed to leukocyte activation. Here, we shine a light on and put forward a unique role of the interleukin (IL)-1 family member IL-18 in experimental APAP-induced ALI. Indeed, amelioration of disease as previously observed in IL-18-deficient mice was further substantiated herein by application of the IL-18 opponent IL-18-binding protein (IL-18BPd:Fc) to wild-type mice. Data altogether emphasize crucial pathological action of this cytokine in APAP toxicity. Adding recombinant IL-22 to IL-18BPd:Fc further enhanced protection from liver injury. In contrast to IL-18, the role of prototypic pro-inflammatory IL-1 and tumor necrosis factor-α is controversially discussed with lack of effects or even protective action being repeatedly reported. A prominent detrimental function for IL-18 in APAP-induced ALI as proposed herein should relate to its pivotal role for hepatic expression of interferon-γ and Fas ligand, both of which aggravate APAP toxicity. As IL-18 serum levels increase in patients after APAP overdosing, targeting IL-18 may evolve as novel therapeutic option in those hard-to-treat patients where standard therapy with *N*-acetylcysteine is unsuccessful. Being a paradigmatic experimental model of ALI, current knowledge on ill-fated properties of IL-18 in APAP intoxication likewise emphasizes the potential of this cytokine to serve as therapeutic target in other entities of inflammatory liver diseases.

## Introduction

Intended or unintended overdosing of acetaminophen [paracetamol, *N*-acetyl-*p*-aminophenol (APAP)] is regarded a major cause of acute liver failure provoking roughly 50,000 emergency room admissions, 2,500 hospitalizations, and 500 fatalities per year in the United States. The global burden on health-care systems that connects to APAP is based on a narrow therapeutic margin and supported by its broad over-the-counter availability. In fact, adverse consequences of APAP (self-)pharmacotherapy fuel a sustained discussion on safety issues and regulations regarding this fairly weak but frequently used analgesic drug (1–4).

Murine models of APAP-induced acute liver injury (ALI) are well established and adequately resemble key features of human intoxication (5). A crucial characteristic of APAP-induced ALI is centrilobular hepatocyte necrosis driven by *N*-acetyl-*p*-benzoquinone imine (NAPQI), an APAP metabolite generated by hepatic Cyp2e1 and Cyp1a2. Under the influence of NAPQI hepatocytes endure oxidative stress, malfunction of mitochondrial respiration, a drop in ATP, and predominantly necrotic cell death. Here, standard therapy with *N*-acetylcysteine interferes by providing NAPQI detoxifying glutathione (GSH) and by counteracting APAP-associated oxidative stress. Aforementioned noxious chain of events is amplified by cell intrinsic processes, among others activation of c-Jun N-terminal kinase (5–7). Since necrosis, by releasing danger-associated molecular patterns, notoriously connects to activation of innate immunity and inflammation (8), an additional immunological layer is considered a significant parameter determining APAP-induced ALI. Accordingly, several toll-like receptors (TLR) such as TLR4 (9, 10) and TLR3 (11) were reported to aggravate poisoning though conflicting reports impede straightforward interpretation (12, 13). Interestingly, TLR9 recognizing necrotic DNA has been identified consistently as pathogenic factor (12, 14, 15). Accordingly, TLR9 antagonism (16) or suppression of downstream type I interferon (IFN) function can ameliorate APAP toxicity (17, 18).

Whereas sterile liver inflammation is an undisputed consequence of APAP overdosing (5) its function is multifaceted and not fully understood. In this context, it must be appreciated that outcome of APAP poisoning is, to a significant degree, dependent on the capacity of the liver to activate repair and regeneration processes. Notably, in the later regenerative phase of intoxication, growth factors such as epidermal growth factor receptor ligands (19) but also pro-regenerative signal transducer and activator of transcription (STAT)-3-activating cytokines are regarded pivotal for efficient organ recovery (20). An interesting case in this context is interleukin (IL)-22 (21). Genetic models using *IL22BP*-deficient mice suggest a pathogenic role for endogenous IL-22 particularly during early intoxication (22). In contrast, administration of a single supra-physiological dose of recombinant IL-22 or its provision by liver-targeted IL-22 gene therapy mediates significant protection against APAP-induced ALI (23–25).

It adds to the overall complex nature of APAP-induced ALI that just sterile inflammation appears to be a prerequisite for activation of an efficient hepatic pro-regenerative program (20, 26).

## A Complex Role for Paradigmatic Nuclear Factor (NF) κB-Activating IL-1 And TNFα In APAP-Induced ALI

Sterile inflammation is largely initiated by NF-κB-activating cytokines among which IL-1 and TNFα stand out as crucial (8, 27–29). Whereas hepatic upregulation of IL-1α/β during APAP-induced necroinflammation is undisputed (14, 30–32), the contribution of IL-1 to disease is undecided on every level of IL-1 biology. For example, inhibition of IL-1β maturation by lack of caspase-1 activity in C57BL/6 mice left APAP-induced ALI either unaffected (32, 33) or significantly bettered disease outcome (14). Notably, although IL-1α is not a caspase-1 substrate its protease activity is required for effective IL-1α release by monocytes (34). IL-1α/β-unresponsive IL-1 receptor-1 (IL-1R1)-deficient C57Bl/6 mice likewise displayed discordant behavior with either no effect (30) or amelioration of APAP intoxication (32, 35) put on record. Finally, administration of IL-1 receptor antagonist (IL-1Ra) (36) or neutralizing antibodies targeting either IL-1α (32) or IL-1β (14) improved APAP-induced ALI in C57Bl/6 mice. Surprisingly, IL-1Ra-deficient mice also displayed weakened intoxication (37), though BALB/c mice were used in that study. Alike IL-1, TNFα is evidently produced during APAP-induced ALI (11, 23, 38) and similarly puzzling with regard to function. Exemplarily, a report using TNFα-neutralizing antibody-treated or TNF receptor-1-deficient BALB/c mice proposed pathological action of this cytokine (39). Others found that TNFα-neutralization likewise inhibits (40) or is unable to influence (41) APAP toxicity in C57Bl/6 mice. Surprisingly, TNF receptor-1-deficiency actually aggravated disease in this mouse strain (42).

Differences in mouse characteristics, including the microbiome (43), as well as variations in APAP dosing may foster divergent conclusions regarding the role of IL-1 and TNFα in APAP-induced ALI. However, those observations may also echo an overlapping double-edged function of inflammation in the context of APAP overdosing. Specifically, while inflammation may initially promote early injury, hepatic repair and regeneration in a later phase of disease apparently rely on signals derived from innate immunity and associated cytokines. IL-1 and TNFα, for example, are able to upregulate pro-regenerative IL-6 as well as antioxidant pathways that enforce repair (20, 42, 44, 45). Some previous studies actually indicated a protective role of endogenous IL-1 (37) and TNFα (42) in APAP-induced ALI. Moreover, pro-inflammatory IL-36γ (46) was recently shown to promote regeneration in APAP toxicity, an observation that agrees with IL-36 supporting intestinal repair (47, 48). It is noteworthy that an early study reported on amelioration of APAP-induced ALI by application of recombinant IL-1α (49).

## A Distinctive Role for IL-18 in APAP-Induced ALI

Due to some unique properties, IL-18 stands out among members of the IL-1 cytokine family (50, 51). IL-18 is constitutively expressed in a variety of cell types, for example, in hepatic Kupffer cells (52). Accordingly, IL-18 expression is detectable in healthy murine liver (53) where macrophages/Kupffer cells are a major source of bioactive IL-18 (50, 51). The active processed cytokine is usually (but not exclusively) generated by caspase-1 upon inflammatory stimulation (54). Besides being an inflammatory NF-κB-activating cytokine (55–57), two exceptional characteristics are key to the function of IL-18 in liver diseases. First of all, IL-18, initially coined IGIF for IFNγ-inducing factor (58), is pivotal for IFNγ production by T (58) and natural killer (NK) cells (59). In addition, IL-18 is a strong inducer of Fas ligand (FasL), particularly on NK cells (60). Both characteristics should be of significance for APAP-induced ALI because IFNγ (31) and Fas/FasL signaling (38, 61, 62) are crucial for the development of full APAP toxicity.

**Figure 1:**
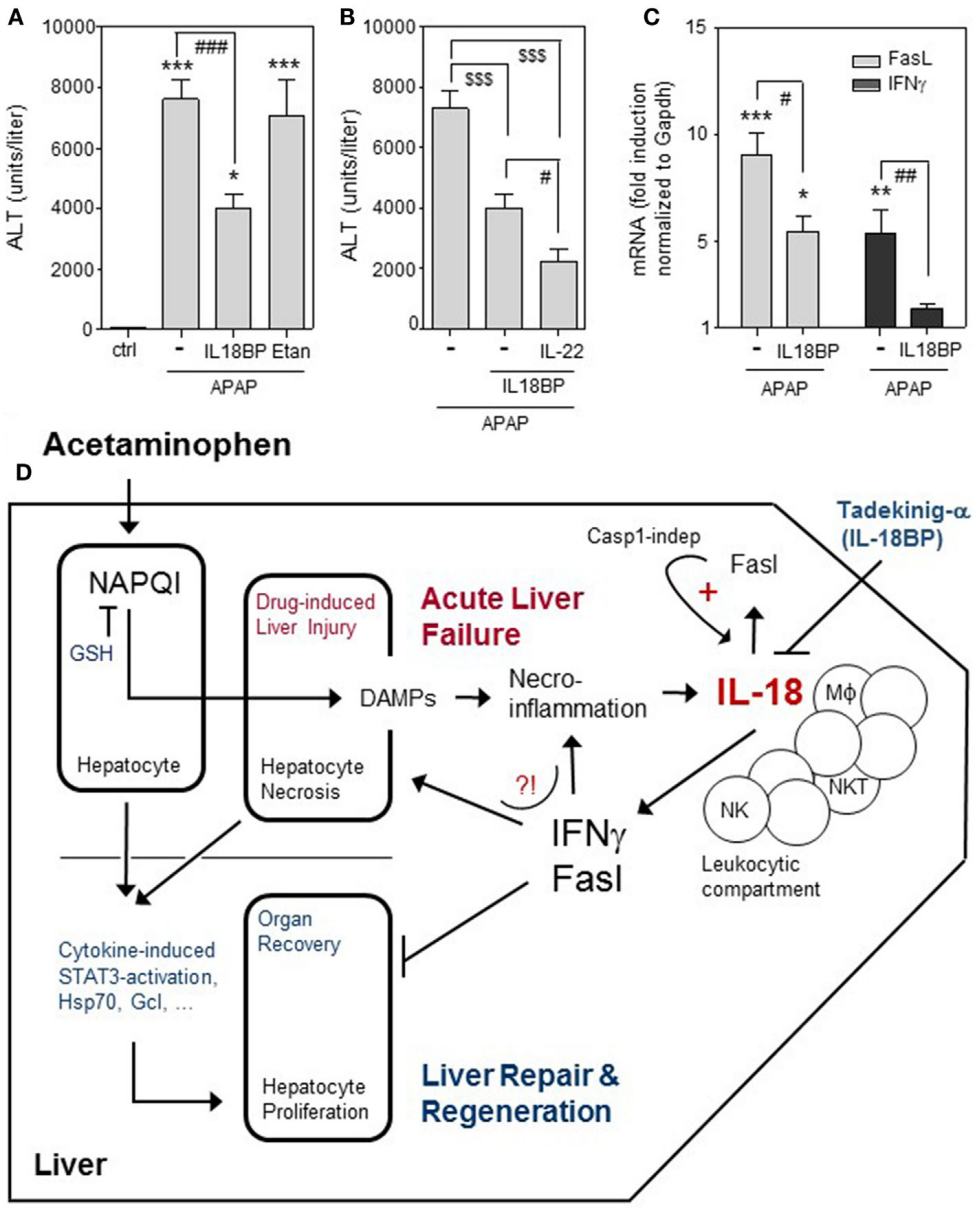
Effects of IL-18BPd:Fc on APAP-induced ALI. **(A–C)** All animal experiments (fasted male C57Bl/6 mice, 9–10 weeks old) were carried out in accordance with the recommendations of the Animal Protection Agency of the Federal State of Hessen (Regierungspräsidium Darmstadt, Germany). The protocol was approved by the Regierungspräsidium Darmstadt (Germany). The model of murine APAP (i.p. 500 mg/kg in 0.9% NaCl)-induced liver injury was performed as recently described (46). Where indicated, mice were i.v. cotreated with recombinant IL-18BPd:Fc (IL18BP), IL-22, or etanercept in PBS. **(A)** Mice received either APAP (*n* = 18), APAP/IL-18BPd:Fc (15 µg, *n* = 12), APAP/Etanercept (75 µg, *n* = 7), or 0.9% NaCl/PBS (ctrl, *n* = 6). After 24 h, serum alanine aminotransferase (ALT) activity was determined (Reflotron, Roche Diagnostics, Mannheim, Germany) and is depicted as units/liter (means ± SEM). *P < 0.05, ***P < 0.001 compared to ctrl; ^###^*P* < 0.001. **(B)** Mice received either APAP/PBS (*n* = 12), APAP/IL-18BPd:Fc (15 µg; *n* = 12), or APAP/IL-18BPd:Fc (15 µg) plus IL-22 (3.5 µg) (*n* = 13). After 24 h, serum ALT activity was determined and is depicted as units/liter (means ± SEM). ^$$$^*P* < 0.001, ^#^*P* < 0.05. **(C)** Mice received either APAP/PBS (*n* = 12) or APAP/IL-18BP (15 µg, *n* = 12) and were maintained for 16 h. For RNA analysis, liver tissue was snap frozen in liquid nitrogen and stored at −80°C. Total RNA was isolated as described (18). For real-time PCR, pre-developed reagents were used (Thermo Fisher Scientific, Darmstadt, Germany): GAPDH (VIC; 4352339E), Fas ligand (FasL) (FAM; Mm00438864_m1), and IFNγ (FAM; Mm01168134_m1). Assay mix was from Nippon Genetics (Düren, Germany). PCR: one initial step at 95°C (2 min) was followed by 40 cycles at 95°C (5 s) and 62°C (30 s). Detection of the dequenched probe, calculation of threshold cycles (*C*_T_ values), and data analysis were performed by the Sequence Detector software (AbiPrism7500 Fast Sequence Detector, Thermo Fisher Scientific). Relative changes in hepatic FasL [**(C)**, left panel] and IFNγ [**(C)**, right panel] mRNA expression determined by real-time PCR were normalized to that of GAPDH and shown as fold-induction compared with untreated control mice (*n* = 6). *P < 0.05, **P < 0.01, ***P < 0.001 compared with untreated control; ^#^*P* < 0.05, ^##^*P* < 0.01. **(A–C)** Data are shown as means ± SEM. Raw data were analyzed by one-way ANOVA with *post hoc* Bonferroni correction. **(D)** Graphical summary of processes affecting outcome of APAP-induced ALI with focus on the pathogenic role of IL-18. Detrimental pathways activated by APAP overdosing are counteracted by endogenous mechanisms supporting organ recovery through repair and regeneration [e.g., hepatocyte STAT3 activation; expression of heat shock protein (Hsp)70 and glutamate-cysteine ligase (Gcl)]. If therapeutic NAC intervention aiming at augmentation of hepatocyte glutathione (GSH) fails due a to an exceedingly high APAP dosage, a too late time point of intervention, and/or a pre-damaged liver parenchyma, acute liver failure may proceed to an ill-fated condition requiring transplantation for patient survival. Here, IL-18 may play a unique role by supporting hepatic expression of FasL and IFNγ. Application of recombinant IL-18 binding protein (Tadekinig-a) may evolve as a novel therapeutic option to intervene at this point.

In accord with aforementioned characteristics, IL-18-deficient mice display strong protection from APAP-induced ALI (14). Since the pathogenic role of IL-18 in APAP intoxication has, best to our knowledge, not been confirmed in wild-type mice, we set out to determine consequences of IL-18 neutralization in this context. APAP (500 mg/kg) was applied intraperitoneally to fasted male C57Bl/6 mice as previously described (46). Where indicated, mice were i.v. cotreated with recombinant murine IL-18BPd:Fc (15 μg/mouse, R&D Systems, Wiesbaden, Germany). This genetically engineered agent corresponds to the neutralizing murine IL-18 opponent IL-18 binding protein d (IL-18BPd) (50, 63). Liver injury was quantified by determining serum alanine aminotransferase (ALT) activity 24 h after APAP administration, a time point coinciding with maximal hepatic damage in this protocol (46). In accord with data on IL-18-deficient mice, blockage of murine IL-18 biological activity by IL-18BPd:Fc indeed improved APAP-induced ALI (Figure 1A). As already alluded to, we and others have previously reported on amelioration of APAP intoxication by therapeutic provision of IL-22 (23–25). Interestingly, adding IL-22 (i.v. 3.5 μg/mouse, Immunotools, Friesoythe, Germany) to IL-18BPd:Fc further diminished serum ALT activity with an overall reduction by 69.5 ± 5.8% (Figure 1B). As further control, mice were i.v. treated with etanercept (75 μg/mouse, Pfizer, Karlsruhe, Germany), a clinically used TNFα blocker (TNFR2:Fc) known to likewise neutralize biological activity of the murine cytokine (64). As shown in Figure 1A, TNFα blockage did not affect APAP toxicity. Altogether, we confirm previous observations on a pathogenic role of IL-18 (14) and on lack of TNFα function (41) in APAP-induced ALI.

The pathogenic role of IL-18 during APAP-induced ALI likely connects to the aforementioned potential to upregulate hepatic IFNγ and FasL. Both latter parameters are increased in liver tissues of APAP-challenged mice (38). Administration of IL-18BPd:Fc in fact suppressed hepatic expression of FasL (Figure 1C, left panel) and IFNγ (Figure 1C, right panel) in APAP-treated mice. Interestingly, IFNγ is known to support hepatocyte necrosis in response to APAP, possibly by enhancing nitric oxide formation (5, 31). IFNγ may additionally impair APAP-associated liver regeneration (45). This detrimental IFNγ activity has been shown to determine course of disease in experimental partial hepatectomy (65). The pathogenic role of Fas/FasL in APAP-induced ALI is likewise well established, detectable in Fas- or FasL-deficient (38, 62) as well as in wild-type mice (61), and apparently mediated by non-canonical Fas action. Specifically, apoptosis of hepatocytes is not regarded as relevant mechanism contributing to APAP-induced ALI. Accordingly, hepatocyte apoptosis by Fas/FasL is largely ruled out as relevant pathogenic mechanism in that context (26). Although Fas is famous for mediating apoptosis, it is noteworthy that this receptor can also activate classical signal transduction, e.g., *via* mitogen-activated protein kinases and NF-κB (66) which disconnects from pro-apoptotic signaling (67). Pathogenic action of Fas in APAP-induced ALI has been related to downregulation of glutamate-cysteine ligase and prolongation of GSH depletion as well as to reduction of heat shock protein (HSP)-70 (62). HSP70 is protective in APAP poisoning (68) and actually supports liver regeneration in murine partial hepatectomy (69). Moreover, Fas deficiency connects to impaired expression of STAT3-activating IL-6 and IL-10 (62), both are capable of ameliorating APAP-induced ALI (20). It is a further remarkable facet that interactions between hepatic macrophages and lymphocytes directed by Fas/FasL actually support production of bioactive IL-18 in caspase-1-independent but caspase-8-dependent manner (70, 71). Since IL-18 enhances FasL expression (60) which in turn enhances IL-18 (70, 71) this regulatory path represents a classical vicious cycle promoting liver pathology (54). Figure 1D provides a graphical summary of the complex events affecting outcome of APAP-induced ALI with focus on the pathogenic role of IL-18.

## Concluding Remarks

The unresolved role of NF-κB-activating inflammatory cytokines including that of the caspase-1/IL-1β axis in APAP-induced ALI (20, 26, 72–74)—see Table 1—may reflect Janus-faced properties of theses mediators in the early injury and the later (partly overlapping) regeneration phase of intoxication. Herein, we confirm and put forward the perspective that IL-18 plays a unique pathogenic role in this model of sterile inflammation. Regardless of whether being activated by caspase-1, caspase-8, or by extracellular proteases such as proteinase-3 (50, 54), the potential of mature IL-18 to upregulate hepatic IFNγ and FasL appears decisive for its function during APAP-induced ALI. It is noteworthy that a detrimental role for hepatic IL-18 is not only conceivable for APAP intoxication. Specifically, administration of IL-18 neutralizing antibodies or recombinant IL-18 binding protein likewise ameliorates *Propionibacterium acnes*/lipopolysaccharide- (53, 75) as well as concanavalin A-, Fas-, and *Pseudomonas aeruginosa* exotoxin A-induced murine liver damage (75). Moreover, treatment with recombinant IL-18 binding protein protected from liver injury in murine experimental hemophagocytic lymphohistiocytosis (76). Current data also suggest an additional benefit of the combination IL-18BPd:Fc plus IL-22, an observation that deserves delineation in forthcoming experiments.

**Table 1:** Data on the role of IL-18, IL-1, caspase-1, and TNFα in experimental APAP-induced ALI as detected in C57Bl/6 and BALB/c mice.

IL-18 blockage	↓ IL-18BPd:Fc (herein); ↓ *il18*^−/−^ mice (14)
IL-1-blockage	≈ *il1r1*^−/−^ mice (30)
	↓ *il1r1*^−/−^ mice (32, 35); ↓ anti-IL-1β (14); ↓ anti-IL-1α (32)
IL-1 receptor antagonist deficiency	**↓** *il1ra*^−/−^ mice (37), using BALB/c mice
Casp-1 blockage	≈ *casp1*^−/−^ (32, 33)
	↓ *casp1*^−/−^( (14)
TNFα blockage	≈ Etanercept (herein); ≈ anti-TNFα (41)
	↑ TNF-R-*p55*^−/−^ (42)
	↓ anti-TNFα (39) using BALB/c mice (40)
	↓ TNF-R-*p55*^−^/( [(39) using BALB/c mice]

*Unless otherwise indicated, data were generated using C57Bl/6 mice*.

*Casp-1, caspase-1; ≈, lack of effect; ↓, amelioration of disease; ↑ aggravation of disease*.

Current data altogether advocate short-term blockage of IL-18 as therapeutic approach in acute liver diseases. A recent phase I/II clinical trial investigating application of recombinant IL-18BP (tadekinig-α) in adult onset still’s disease actually revealed an acceptable safety profile of this agent—besides specific therapeutic efficacy (77). Moreover, in human acute liver failure due to APAP overdosing, elevated levels of IL-18 are detectable in patients’ sera (78). It is thus tempting to speculate that provision of interleukin-18 binding protein therapy aids those unfortunate patients where standard therapy with *N*-acetylcysteine falls short.

## Ethics Statement

All animal experiments using C57Bl/6 mice (male, 9–10 weeks old) were carried out in accordance with the recommendations of the Animal Protection Agency of the Federal State of Hessen (Regierungspräsidium Darmstadt, Germany). The protocol was approved by the Regierungspräsidium Darmstadt (Germany).

## Author Contributions

MB: performed all experiments, analyzed the data, and contributed to manuscript writing and editing. JP: analyzed the data and contributed to manuscript editing. HM: analyzed the data, designed the study, wrote the paper, and performed manuscript editing.

## Conflict of Interest Statement

The authors declare that the research was conducted in the absence of any commercial or financial relationships that could be construed as a potential conflict of interest.
